# High Correlation of the Response of Upper and Lower Lobe Small Airway Epithelium to Smoking

**DOI:** 10.1371/journal.pone.0072669

**Published:** 2013-09-09

**Authors:** Ben-Gary Harvey, Yael Strulovici-Barel, Thomas L. Vincent, Jason G. Mezey, Ramya Raviram, Cynthia Gordon, Jacqueline Salit, Ann E. Tilley, Augustine Chung, Abraham Sanders, Ronald G. Crystal

**Affiliations:** 1 Division of Pulmonary and Critical Care Medicine, Department of Medicine, Weill Cornell Medical College, New York, New York, United States of America; 2 Department of Genetic Medicine, Weill Cornell Medical College, New York, New York, United States of America; 3 Department of Biological Statistics and Computational Biology, Cornell University, Ithaca, New York, United States of America; Johns Hopkins School of Medicine, United States of America

## Abstract

The distribution of lung disease induced by inhaled cigarette smoke is complex, depending on many factors. With the knowledge that the small airway epithelium (SAE) is the earliest site of smoking-induced lung disease, and that the SAE gene expression is likely sensitive to inhaled cigarette smoke, we compared upper *vs.* lower lobe gene expression in the SAE within the same cigarette smokers to determine if the gene expression patterns were similar or different. Active smokers (n = 11) with early evidence of smoking-induced lung disease (normal spirometry but low diffusing capacity) underwent bronchoscopy and brushing of the upper and lower lobe SAE in order to compare upper *vs* lower lobe genome-wide and smoking-responsive gene expression by microarray. Cluster and principal component analysis demonstrated that, for each individual, the expression of the known SAE smoking-responsive genes were highly correlated in upper and lower lobe pairs, although, as expected, there were differences in the smoking-induced changes in gene expression from individual to individual. These observations support the concept that the heterogeneity observed among smokers in the anatomic distribution of smoking-induced disease are not secondary to the topographic differences in the effects of cigarette smoke on the airway epithelium.

## Introduction

The human airways are complex, dichotomous branching structures with up to 2^23^ branches from the trachea to the alveoli [1]. Extensive morphologic and, more recently, imaging analysis has emphasized regional heterogeneity in number, length, width and branching angles of the airways in the upper *vs* lower lung zones [2], [3]. This, together with upper *vs* lower lobe differences in blood flow, effects of gravity and differential effects of pressure-volume relationships modulate the differences in volume and flow of inspired air to the upper *vs* lower lobes [4]–[6]. These topographic upper *vs* lower lobe differences are relevant to how gases, xenobiotics and particulates suspended in inhaled air impact the airways to varying degree, depending on their size, charge and other properties [7], [8].

The classic example of these concepts relates to cigarette smoke, a complex mixture of gases and particulates [9], [10]. While the smoking-induced lung diseases emphysema and lung cancer exhibit anatomic heterogeneity [2], [3], [11]–[13], it is not clear whether this is secondary to the skewed anatomic distribution of inhaled cigarette smoke or whether this reflects increased susceptibility of some anatomic regions to these diseases [14]. The dispersion of cigarette smoke within the lung is very complex, dependent on individual inhalation parameters, breath holding, the properties of the individual components of cigarette smoke and the differences in lung structure and function among smokers [7], [15]–[17].

Based on the concept that the small airway epithelium (SAE) is the earliest site of smoking-induced lung disease [18]–[21], we have approached the question of topographic distribution of cigarette smoke *vs* topographic susceptibility by comparing the patterns of gene expression in the SAE of the upper *vs* lower lobes in the same cigarette smokers. Studies from our laboratory [22]–[26] and others [27], [28] have shown that cigarette smoking induces marked changes in the airway epithelial gene expression program of clinically healthy individuals. From our prior studies of occasional smokers and smokers exposed to second hand smoke, we know that the SAE is exquisitely sensitive to cigarette smoke, in that if nicotine or its derivatives can be detected in urine at any level, the SAE will exhibit up- and down-regulation of smoking-related genes [25]. In that context, the SAE is a sensitive “canary” that detects cigarette smoke and responds with changes in gene expression that are proportional to the extent of exposure to cigarette smoke up to a certain level of smoking, at which point the SAE gene expression response to smoking “saturates”, i.e., for active smokers, additional smoking does not enhance the response [25]. However, we also know that for active smokers, the response to smoking is variable among individuals in regard to the number of small airway epithelial genes responding to active cigarette smoking, i.e., while smokers as a group have changes in the SAE expression of hundreds of genes, some smokers have little response to smoking, while others respond with marked changes in gene expression. Thus, each smoker needs to be assessed individually.

With this background, we have used the sensitivity of the SAE to cigarette smoke to ask: what is the topographic impact of cigarette smoking on the upper *vs* lower lobe SAE in humans who smoke? To accomplish this, we assessed the impact of cigarette smoking on genome-wide and smoking-responsive gene expression in the SAE of the upper *vs* lower lobes of the same individuals, all with early manifestations of smoking-induced disease evidenced by normal spirometry, but low diffusing capacity (DLCO), i.e., smokers considered “normal” by the standard criteria for chronic obstructive pulmonary disease (COPD) [29], but with some evidence of smoking-related abnormal lung function.

## Methods

### Study Population

The protocol and informed consent for this study were approved by the Weill Cornell Medical College Institutional Review Board. Current smokers were recruited from the general population in New York City and written informed consent obtained. Individuals were evaluated at the Weill Cornell NIH Clinical and Translational Science Center and the Department of Genetic Medicine Clinical Research Facility. Each had a normal history, physical exam, complete blood count, coagulation studies, liver function tests, urine studies, chest X-ray, EKG and pulmonary function tests, except for an isolated reduction in DLCO (<80% predicted). All were negative for HIV1 and had normal α1-antitrypsin levels (see Methods S1 for full inclusion/exclusion criteria).

### Chest Computed Tomography Scans

Chest high resolution computed tomography (HRCT) scans were used to determine the percentage of lung affected by emphysema in each of the subjects. The lung was divided into quartiles by lung volume, and the top and bottom quartiles were compared for % emphysema at −950 Hounsfield Units (HU). See Methods S1 for details regarding quantification of emphysema in the scans.

### Upper and Lower Lobe Small Airway Epithelial Sampling

SAE (10^th^ to 12^th^ generation) was collected using flexible bronchoscopy as previously described [23]. The right lower lobe was brushed first, immediately followed by brushing of the right upper lobe. Cells from the two sources were processed separately (see Methods S1 for details). RNA was hybridized on Affymetrix HG-U133 Plus 2.0 arrays (Affymetrix Inc., Santa Clara, CA) with probes for >54,000 genome-wide transcripts, using Affymetrix protocols, hardware and software [30]. Microarray quality was verified by GAPDH 3′ to 5′ probe sets <3.0 and multi-chip normalization scaling factor <10.0 [31]. The raw data is publically available at the Gene Expression Omnibus (GEO) site (http://www.ncbi.nlm.nih.gov/geo/), accession number GSE26307.

### Assessment of Gene Expression

Data were normalized using the MAS5 algorithm (Microarray Suite Version 5, GeneSpring version 7.3, Affymetrix) per chip to the median expression value of each sample and per gene to the median expression value of each gene across all samples. Genome-wide analysis was used to compare the expression in the upper *vs* lower lobe of the same individual. We performed a paired limma test (linear model for microarray data) to identify differentially expressed genes between upper and lower lobe samples [32]. Limma fits a linear model to the expression data of each gene and uses an empirical Bayes approach to shrink the sample variance towards a common estimate. This has the advantage of making the analysis more stable and allows for more robust inference, even for experiments with a small number of samples. In addition, in order to reduce the number of false-negatives due to the low number of samples, we applied a “q-value” method that allows control of the false-discovery rate by controlling for p value distribution [33]–[35]. As an additional statistical test, we applied the SMVar (Structural Model for Variances) method, which also allows implementation of a moderated t-test for paired data. SMVar assumes a linear mixed model on the logarithm of the residual variances to be a function of both gene and condition, therefore allowing us to estimate gene-specific residual variances across the entire dataset [34], [36].

Expression values were compared using 3 lists of probe sets: (1) smoking-responsive probe sets (“smoking-responsive list”, n = 529 probe sets, using the small airway epithelial smoking-responsive gene list of Strulovici-Barel et al [25], corresponding to 372 unique genes); (2) 100 genome-wide random gene lists of 529 probe sets present in at least 20% of the upper or lower lobe samples, smoking-responsive list excluded (“100 random lists”); (3) based on the subjects in this study, we identified smoking-responsive probe sets significantly differentially expressed in smokers with early emphysema *vs* healthy nonsmokers, n = 228 probe sets (comparing 11 smokers with early emphysema *vs* 60 healthy nonsmokers (see Wang et al. [37] for nonsmoker inclusion criteria and demographics), using all probe sets present in at least 20% of the smoker or nonsmoker samples; significantly expressed probe sets were selected using the same criteria used to create the smoking-responsive list, i.e., probe sets with fold-change ≥1.5 between the groups, p<0.01 with Benjamini-Hochberg correction). These genes were functionally annotated using the NetAffx Analysis Center (www.affymetrix.com) to retrieve Gene Ontology (GO) annotations.

### Statistical Analyses

Response of the transcriptomes of the upper and lower SAE was assessed using univariate and multivariate approaches (see Methods S1 for details).

## Results

**Figure 1:**
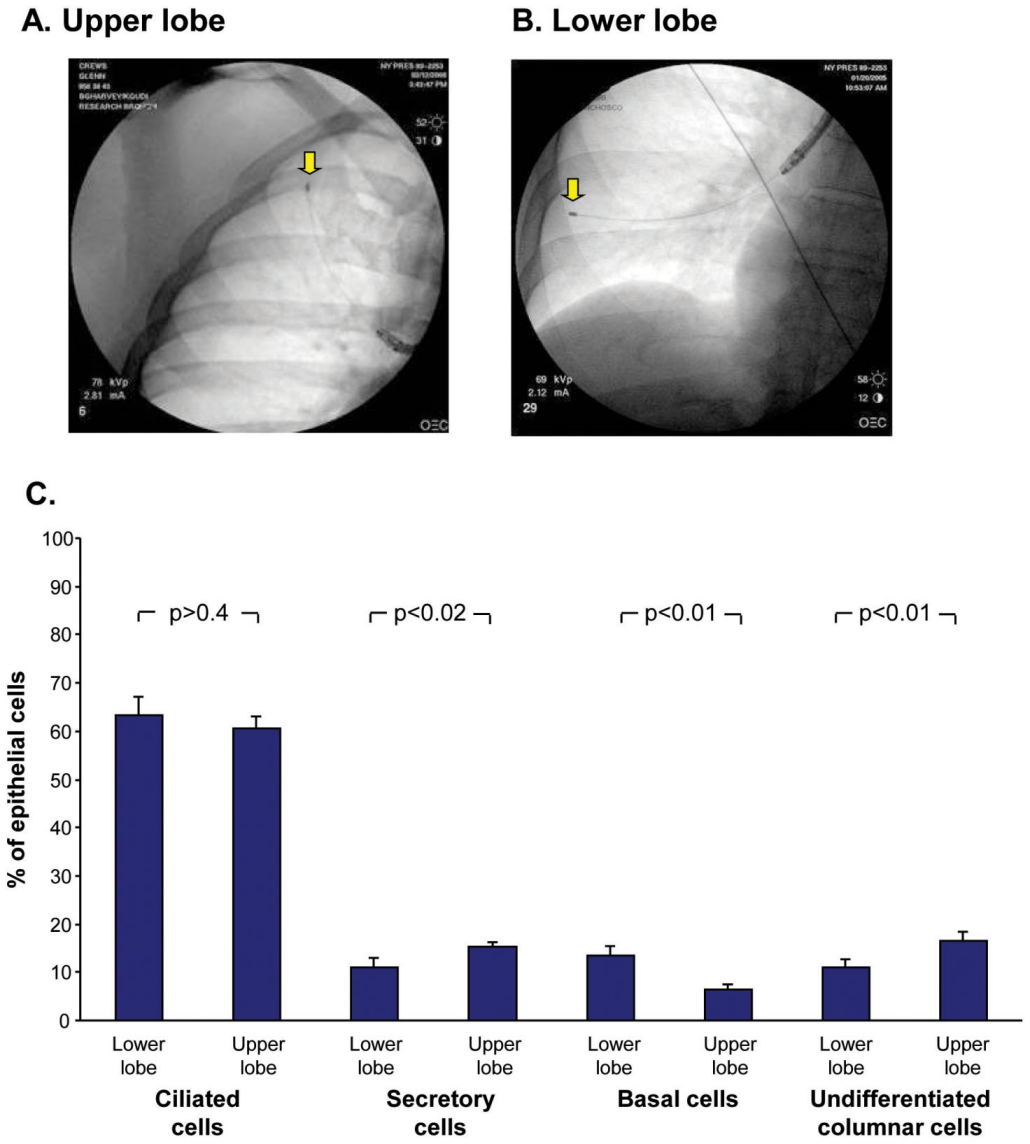
Sampling of small airway epithelium of upper *vs* lower lobes. **A, B.** Fluoroscopy of the upper and lower lobes showing the position of the brush used for sampling (arrows). **C.** Epithelial cell types in the upper *vs* lower lobes. Shown is data (mean ± standard error) based on counting 500 cells in cytocentrifuge preparations of upper *vs* lower lobe small airway epithelial samples. p values for paired t-test between upper and lower lobes are shown.

**Figure 2:**
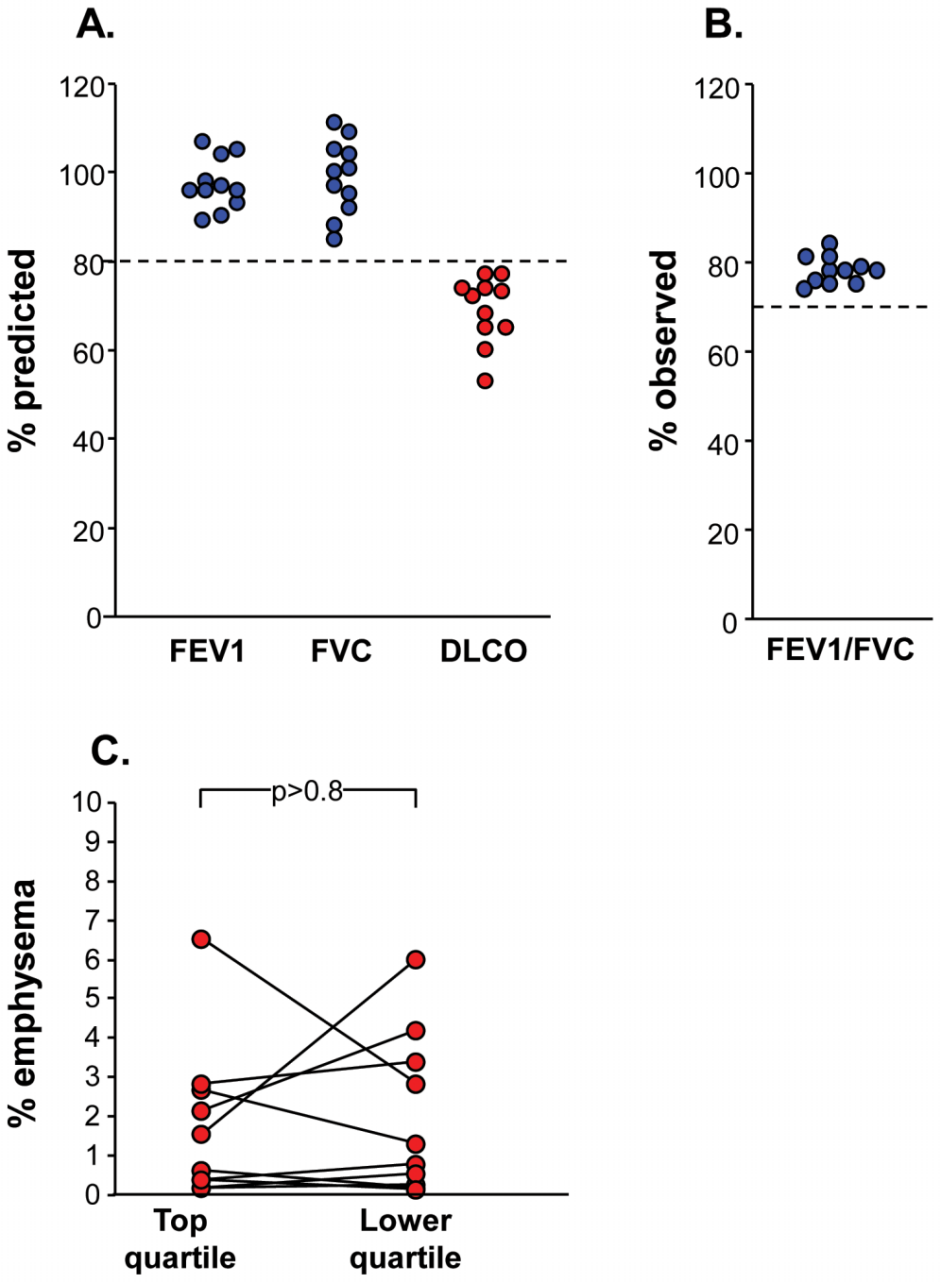
Lung function and chest high resolution computed tomography (HRCT) scans. **A.** Forced expiratory volume in 1 sec (FEV1), forced vital capacity (FVC), and diffusing capacity (DLCO), all as % predicted. **B.** Ratio of FEV1/FVC as % observed. **C.** HRCT quantification of emphysema by −950 Hounsfield Units (HU) divided into top (upper lung zones) and bottom (lower lung zones) quartiles by lung volume.

Small airway epithelial samples were obtained from the upper and the lower lobes of 11 current smokers, all with normal spirometry, but low DLCO (Table I, Figures 1, 2 A,B). All collected samples were comparable in number and purity of the recovered cells. There was no difference in the % ciliated cells recovered, but there were significantly more secretory and undifferentiated columnar cells and less basal cells in the upper *vs* lower lobe (p<0.01, both comparisons, Figure 1C). Quantification of the % emphysema present in the upper *vs* the lower quartiles of the chest HRCT scans for each individual showed overall no significant differences between the upper lobe and the lower lobe % emphysema (p>0.8, Figure 2 C). However, as has been observed by others assessing early disease [12], [13], [38], [39], there was heterogeneity in anatomic distribution among the subjects, with some with upper lobe dominance, others with lower lobe dominance and others with similar upper and lower lobe % emphysema.

**Table 1:** Demographics of the Study Population and Airway Epithelial Samples.^1^

Parameter	Study Population
N	11
Sex (male/female)	7/4
Age (yr)	50±7
Race (B/W/O)^2^	10/1/0
Smoking history (pack-yr)	28±13
Urine nicotine (ng/ml)	666±805
Urine cotinine (ng/ml)	1632±926
Pulmonary function parameters^3^	
FVC	98±10
FEV1	96±9
FEV1/FVC	78±3
TLC	89±13
DLCO	66±9
Epithelial cells^4^– lower lobe	
Number recovered ×10^6^	5.9±2.9
% inflammatory cells	1.1±1.0
% epithelial cells^5^	98.9±0.9
Epithelial cells^4^ – upper lobe	
Number recovered ×10^6^	5.9±2.6
% inflammatory cells	1.0±1.3
% epithelial cells^5^	99.0±1.3

1 Data are presented as mean ± standard deviation.

2 B = Black, W = White, O = Other.

3 Pulmonary function testing parameters are given as % of predicted value with the exception of FEV1/FVC, which is reported as % observed; FVC - forced vital capacity; FEV1 - forced expiratory volume in 1 sec; TLC - total lung capacity; DLCO - diffusing capacity.

4 Small airway epithelium.

5 As a % of small airway epithelium recovered.

### Upper *vs* Lower Lobe Small Airway Gene Expression

Genome-wide analysis was used to compare the gene expression of the SAE in the upper *vs* the lower lobe. The expression levels of all probe sets present in at least 20% of the upper or lower lobe samples (31,883 probe sets) were compared using a paired t-test. After applying a multiple test correction (Benjamini-Hochberg), the paired t-test analysis did not return any significant differences for any probe sets (not shown). In order to compare the genes responding to cigarette smoking in the SAE of the upper *vs* lower lobe, the expression levels of the 529 probe sets in the smoking-responsive list [25] and the 228 probe sets significantly differently expressed between the smokers with low DLCO samples and healthy nonsmoker controls were compared using a paired t-test. After applying a multiple test correction (Benjamini-Hochberg), there were no significant differences in the probe sets for either list (not shown). These results were further validated with analysis using limma and SMVar differential gene expression methods.

**Figure 3:**
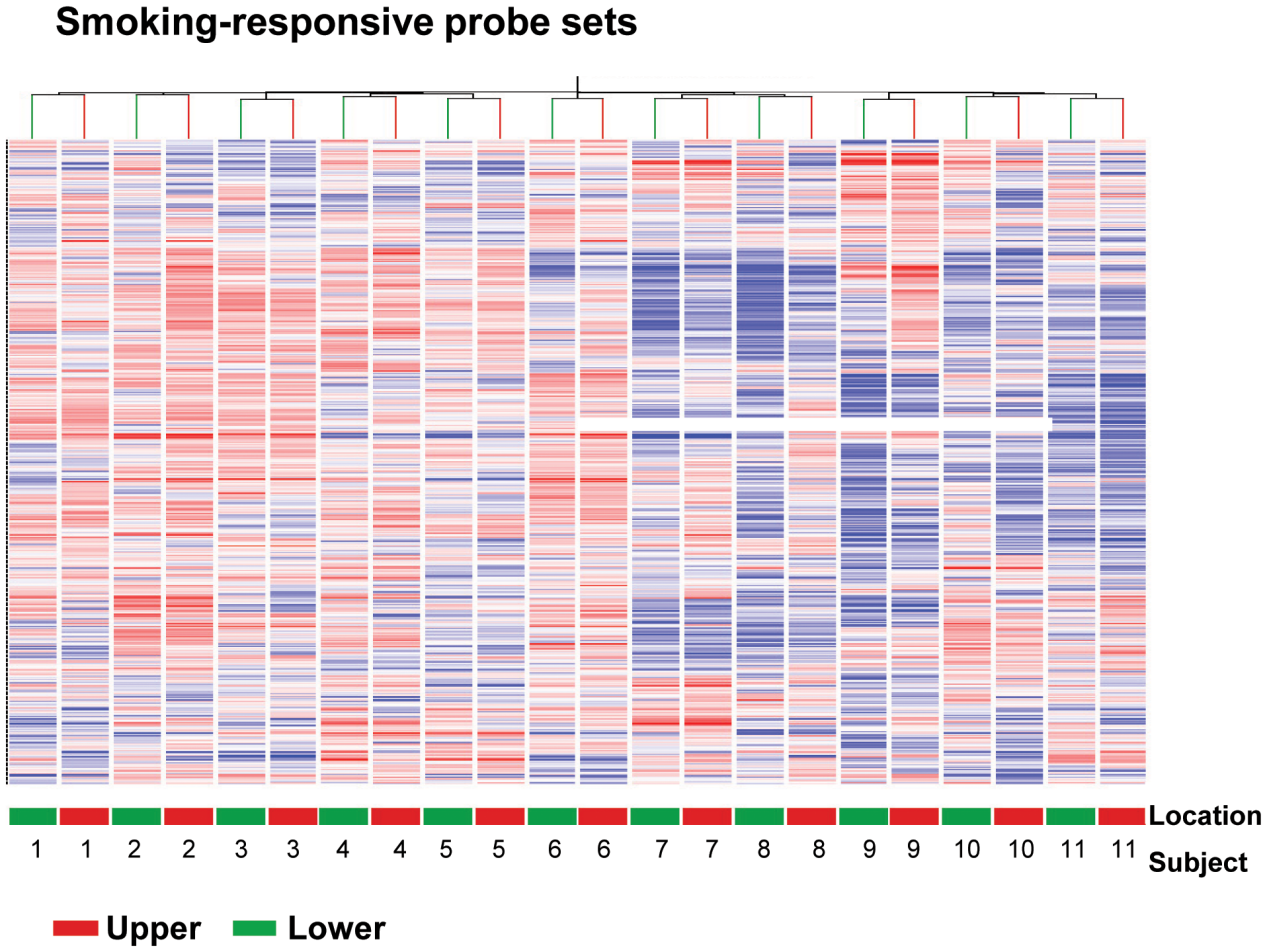
Cluster analysis of upper *vs* lower lobe gene expression to assess if, for each individual, the expression of known smoking-responsive genes is highly correlated in the upper *vs* lower lobes. Shown is a cluster analysis of small airway epithelium 529 known smoking-responsive probe sets corresponding to 372 genes [25]. Note that the smoking-responsive gene expression of the upper *vs* lower lobes for each of the 11 subjects clusters together.

A hierarchical clustering analysis of the 529 smoking-responsive list, using either Pearson or Spearman correlation measures, showed that every pair of smokers (i.e., 11 of 11) clustered together (Figure 3). To assess the significance of returning this many cluster pairs for a set of 529 probe sets, we compared this to the number of cluster pairs for each of the 100 random lists. Using the Pearson or Spearman correlation, none of the random lists produced 11 of 11 pairings (p<0.02, both correlations). These data support the concept that, for the smoking-responsive genes, a sufficient amount of smoke reaches the upper and lower lobes to induce a high correlation of expression of the smoking-responsive genes. Given that the cilia cell population is not significantly different in the upper *vs* lower lobe, which might drive the upper-lower lobe sample pairing, we confirmed that the smoking-responsive gene list did not consist of any cilia-related genes [40]–[42]; therefore, the perfect upper-lower pairing is solely due to the highly correlated SAE gene expression pattern of the smoking-responsive probe sets.

**Figure 4:**
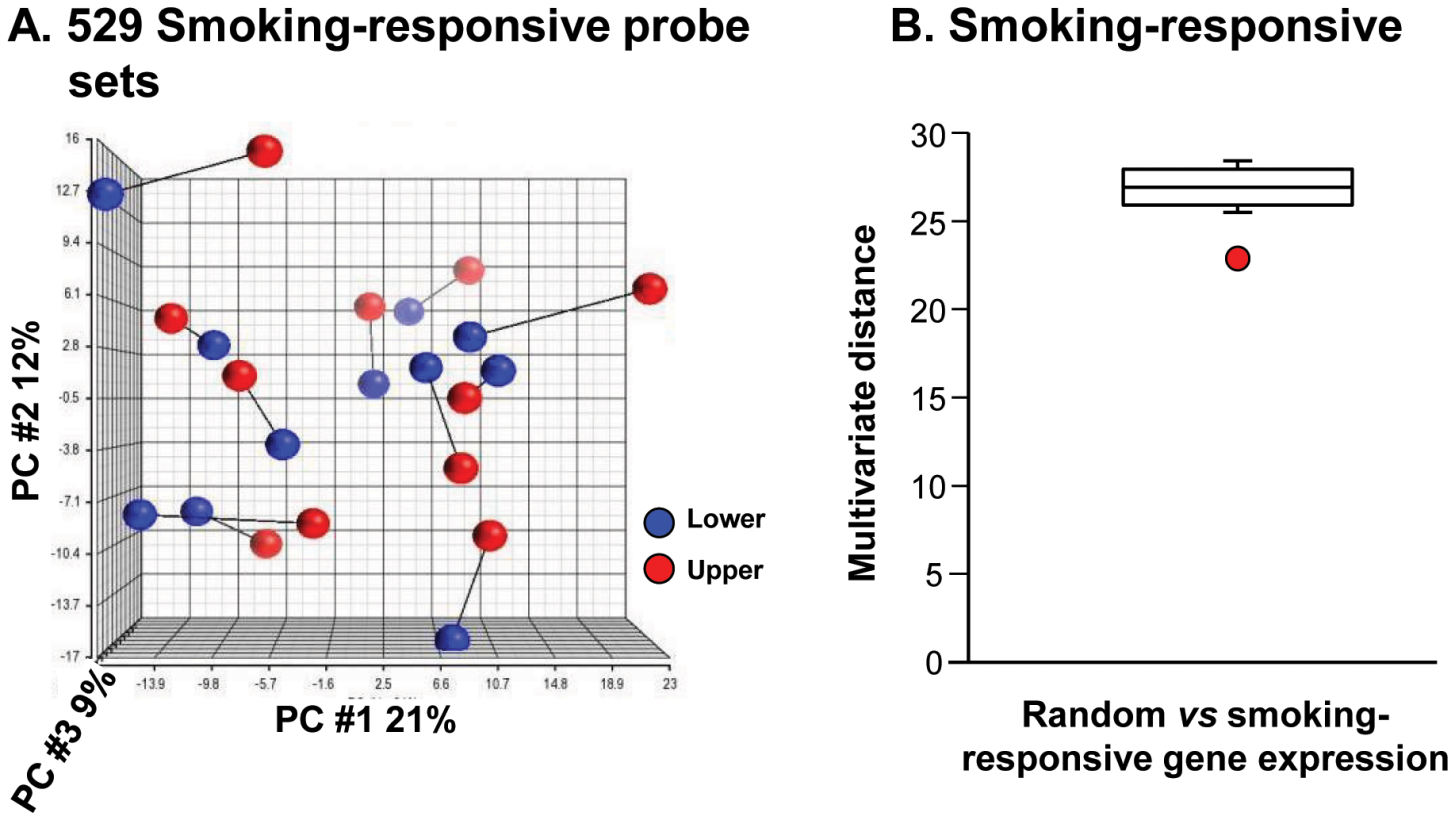
Principal component and multivariate vector length analysis of upper *vs* lower lobe gene expression of known smoking-responsive genes [25]. **A.** Principal component analysis (PCA) of the 529 probe sets of the smoking responsive list. Red = upper lobe samples; blue = lower lobe samples. The line connecting the red and blue data points connects the upper and lower lobe samples from the same individual. **B.** Plot of the distribution of the median distance between the upper and lower lobe samples for each individual in the space of 529 probe sets in the smoking- responsive list as measured by vector length (red circle) compared to the distribution of median distances between upper and lower lobe samples for 100 sets of 529 probe sets selected at random from the genome-wide list of probe sets present in at least 20% of the samples, minus the 529 smoking-responsive list (boxplot: middle bar is overall median, box contains the inner quartiles, and the whiskers, the 0.95 quartiles).

Visual inspection of the first three principal components of the PCA of the smoking-responsive list (Figure 4A) did not explain the high level of pair clustering in the hierarchical analysis, indicating that the pairing was being driven by more variation than captured in the first three components. To assess whether this was the case for the 529 probe sets in this smoking-responsive list, the distance in multivariate probe space between sample pairs was quantified by the length of the vector connecting the upper and lower lobe samples for each individual. The median of these 11 vector lengths for the smoking-responsive list was then compared to the medians of upper and lower sample pairs for 100 random lists (Figure 4B). This analysis showed that the median vector length between each upper and lower lobe pair for the smoking-responsive genes was far less than the median vector length when considering the 100 random lists. Thus, the cluster pairing was being driven by individual smoker overall distance in multivariate probe space and not just the first three principal component dimensions. Together, the cluster and the PCA distance in multivariate probe space support the concept that, for each smoker, the SAE of the upper and lower lobes is responding in a highly correlated way to inhaled cigarette smoke.

## Discussion

The distribution of inhaled cigarette smoke is complex, dependent on a variety of physical, anatomic and physiologic factors, as well as the smoking habits of the individual smokers [4]–[8]. Based on the knowledge that smoking-induced lung disease starts in the SAE [18]–[21], and that smoking induces changes in gene expression in the SAE [23]–[26], we compared the patterns of gene expression in the upper *vs* lower lobes of the SAE of the same cigarette smokers. Interestingly, though there might be proteins preferentially inactivated by smoke in the upper lobe alone, the expression of SAE smoking-responsive genes was remarkably highly correlated in the upper *vs* lower lobes within each smoker, significantly more so than random genome-wide gene expression. This is consistent with the data showing that the SAE is exquisitely sensitive to cigarette smoke at low levels, with proportional increases in smoking-responsive gene expression up to a plateau, where active smokers saturate the small airway epithelial gene expression responses to cigarette smoke [25]. The data suggests that, independent of the complex factors that govern cigarette smoke distribution within the lung, the responses of the SAE to cigarette smoking in humans are highly correlated in the upper and lower lobes, and thus topographic cigarette smoke distribution is not responsible for the topographic differences in smoking-induced disease.

### Airflow in the Human Lung

Studies using radioactive tracers, inhaled particulates, and chest CT scans have shown that distribution of ventilation in the lung is very complex, affected by differences in airway structure, shape and weight of the lung, gravity, posture, and gradients of pleural pressure [43]. In the upright position at rest, the transpulmonary pressure is less at the base and thus the lung at the base is less expanded compared to the apex. Consequently, the lung at the base has greater compliance, and greater relative ventilation. Studies with radioactive gases show that with greater inspiratory flow rate, the regional differences are less than with very slow inspiration [44]. Measurements with xenon CT scans show that ventilation is greater in the central lung and less in the periphery [45]. The effects of gravity on the distribution of ventilation are complicated, with the relative volume of lung in different regions competing with gravity-dependent changes [43]. In addition to the influence of gravity, many studies document that airway architecture also plays a major role in the distribution of air during respiration [4]–[6], [46]–[49]. For example, while branching of airways is invariably dichotomous, it is asymmetric, with the diameter of the 2 branches often unequal, with consequent different flow rates in the 2 branches [43].

### Distribution of Cigarette Smoke

The distribution of the inhaled >4,000 gases, xenobiotics and particulates in cigarette smoke with 10^14^ oxidants/puff is complex, dependent on inhalation parameters of volume and flow, breath holding, the aerodynamic properties of the individual components of smoke and the complex structure and function of the airways in the different regions of the lung [7], [9], [10], [12], [14]. Of the particulates suspended in cigarette smoke, 10 to 90% will be deposited in the airways, depending on aerodynamic diameters [8]. A higher % of particulates are retained in the airways in smokers compared to nonsmokers [8].

The particle size distribution of main stream cigarette smoke has a median diameter of 0.18 to 0.34 µm [14]. Puffing flow rate and duration, interpuff interval and filters all affect the distribution of particles in cigarette smoke [7], [14]. On the average among smokers, there are 8 to 16 puffs/cigarette, 18 to 64 sec between puffs, 232 to 414 sec duration/cigarette, 1.6 to 2.4 sec puff duration, 21 to 66 ml puff volume, 28 to 40 ml/sec peak flow and 413 to 918 ml inhalation volume [17]. There are also differences in the inhalation patterns of smokers [14] as well as gender differences in cigarette puff volume and post-puff inspiratory volume [16]. The consequence of this variability is that there is a wide variation of the topographic distribution of inhaled cigarette smoke within the lung [50]. Other studies found a uniform distribution of ventilation during cigarette smoking [51], [52]. A pathology study assessing the small airways in the upper *vs* lower lobe of smokers with and without emphysema found no difference in inflammation, fibrosis, muscle hypertrophy, squamous metaplasia, intraluminal macrophages and pigment deposition, suggesting a uniform distribution of cigarette smoking [3].

### Topographic Disease in Cigarette Smokers

Lung cancer associated with cigarette smoking tends to be in the upper lung zones, with an upper to lower ratio of 2.5 to 1 [11], [53]. It has been hypothesized that upper lobe predominance is secondary to distribution of cigarette smoke or that cigarette smoke toxins/carcinogens may persist longer in the upper lung zones due to relatively less ventilation or less efficient lymphatic clearance in the upper zones [2]. Another theory is that the upper lobe predominance of lung cancer is due to less efficient delivery of protective substances via the circulation to the upper *vs* lower lung zones [2].

In regard to non-malignant smoking-induced airway and alveolar disease, the data regarding regional differences in disease location is not as clear as with cancer. Morphologic studies demonstrate that the earliest lung abnormalities associated with smoking are in the SAE [18]–[21]. Quantitative morphologic studies have shown that the small airway disease associated with smoking is similar in the upper *vs* lower lung zones [3]. This is consistent with the observations in the present study that the smoking-induced changes in gene expression in the SAE are more highly correlated than random genes in the upper and lower lung zones.

In the later stages of smoking-related non-malignant lung disease, the data is variable, but suggests there is significant heterogeneity in the anatomic distribution of disease. For example, in autopsy cases representing late stage of disease, quantitative morphometry demonstrated upper lobe predominance [38]. In chest X-ray assessment of emphysema, the classic concept is that there is upper lobe predominance, but this conclusion may be, in part, because emphysema is more obvious in the upper lung zones because there is less tissue in the upper lobes [54].

The application of chest CT technology to this issue suggested no differences in the average % emphysema among different lung zones, but recognized there was heterogeneity among subjects, with some with upper lung predominance and others with primarily lower zone emphysema [13]. Consistent with the concept of heterogeneity in the anatomic location of emphysema, others have found homogenously distributed emphysema to be more common than upper or lower zone dominant emphysema [39]. In the National Emphysema Treatment Trial, emphysema was approximately 2 to 1 more prominent in the upper lobes [55]. In contrast, in the Dutch-Belgian Lung Cancer Screening Trial, for 72% of subjects, the topographic distribution was 2.5 to 1 more prominent in the lower lung zones, with 28% of subjects having a more even distribution [15]. More recently, de Torres et al [12], showed that 50% of the tested subjects has homogenously distributed emphysema; these individuals had milder disease than those with upper lung zone predominance. The reasons for the differences in these studies are likely complex, but the overall concept is that there is significant heterogeneity in the anatomic distribution of smoking-related emphysema, with the early disease more homogenous, and the later stages of the disease developing anatomic heterogeneity, with a subset with upper lobe predominance, others with lower zone predominance, and some remaining heterogeneous. This conclusion is consistent with the HRCT data in the present study which showed that, for smokers with mild disease, quantitative HRCT assessment shows heterogeneity in anatomic distribution.

### Topographic Homogeneity of Airway Gene Expression in Cigarette Smokers

Despite the difference in the cell population recovered from the upper *vs* lower lobes in the present study, we observed that the expression of smoking-responsive genes in the SAE of the upper and lower lobes of smokers with mild lung disease is highly correlated. This is consistent with the data of Hackett et al [56] showing that, other than for a few “rapid response” genes that differentially respond to the timing of sample recovery, there was a similar pattern of gene expression in the right *vs* left lower lobe large airway epithelium. The observation that there are no topographic differences in lung gene expression in cigarette smokers was also described in a study of whole lung samples of nonsmokers, ex-smokers and current smokers, concluding that variance among individuals was far greater than between lobes of the same individuals [57].

## Supporting Information

Methods S1 **Supplemental Methods and References.**
(DOC)
