# Supplementary material for: High Correlation of the Response of Upper and Lower Lobe Small Airway Epithelium to Smoking
Source: PLoS One. 2013 Sep 9;8(9):e72669. doi: 10.1371/journal.pone.0072669 (PMC3767732; doi:10.1371/journal.pone.0072669)
Supplement: Methods S1 — Supplemental Methods and References. (DOC) [file pone.0072669.s001.doc]

**S1 Methods**

**Inclusion / Exclusion Criteria for the Study Population**

**Smokers with normal spirometry but low DLCO**

**Inclusion criteria**

1. Males and females, at least 18 years old
2. Capable of providing informed consent
3. Willingness to participate in the study
4. Good health without history of chronic lung disease, including asthma, and without recurrent or recent (within 3 months) acute pulmonary disease
5. Normal physical examination
6. Normal routine laboratory evaluation, including general hematologic studies, general serologic/immunologic studies, general biochemical analyses, and urine analysis
7. Normal PA and lateral chest X-ray
8. Normal electrocardiogram (sinus bradycardia, premature atrial contractions are permissible)
9. Females - not pregnant
10. No history of allergies to medications used in the bronchoscopy procedure
11. Not taking any medications relevant to lung disease or having an effect on the airway epithelium
    - 1. Normal serum α1-antitrypsin level
12. HIV1 negative
13. Self-reported current daily smokers with any number of pack-yr, validated by urine nicotine >30 ng/ml and/or cotinine >50 ng/ml
14. Normal FEV1 (≥80% predicted), FVC (≥80 predicted), FEV1/FVC (≥0.7) based on post-bronchodilator spirometry, TLC (≥80% predicted)
    - 1. DLCO <80% predicted

**Exclusion criteria**

- Unable to meet the inclusion criteria
- Alcohol or drug abuse within the past 6 months
- Evidence of malignancy within the past 5 years
- Current active infection or acute illness of any kind

**High Resolution Computed Tomography Scan Parameters**

Prior to the scan, each individual was asked to take 5 deep breaths and then a slow inspiration to maximum inspiration. The scan time was approximately 8 sec. Reconstruction was performed at 1.25 mm bone and standard algorithm. Using a standard non-contrast protocol in the supine position, the scan was from apex to posterior costophrenic angle 120 kv, 1625 mA, collimation 1.25 mm, volume coverage 27.5 mm/rotation, rotation time 0.8 sec, tube current x rotation time 130 mA, pitch 1.375:1 and image acquisition time 8 sec. Each read was normalized to air and water [1,2].

Analysis of the scans was performed with the EmphylxJ software application (www.icapture.ubc.ca). This application allows for automated quantitative analysis of transverse chest CT scans that are stored in DICOM 3.0 format. After the DICOM images were uploaded, automatic segmentation was used to identify the right and left lung in each slice, and excluding the major airways, blood vessels, mediastinum, and chest wall from the analysis. This segmentation process was supervised by a trained observer, and performed with three passes to optimize the regions identified. When necessary, manual segmentation was performed to correct mistakes made by the automated process. Once segmentation was complete, quantitative analysis was performed by density mask analysis, and a frequency histogram for CT values of -950 Hounsfield units (HU) was generated. Technically satisfactory scans were available for 10 out of the 11 subjects. The lung was divided into quartiles by lung volume, and the top and bottom quartiles compared for % emphysema at -950 HU.

**Small Airway Epithelium Cell Processing**

The cells were removed from the brush by flicking into 5 ml of ice-cold LHC8 medium (GIBCO, Grand Island, NY), with 4.5 ml immediately processed for RNA extraction, and 0.5 ml to determine the number and types of cells recovered as assessed by cytopreparations. The expression of genes encoding Clara cell secretory protein were used to confirm the samples were small airway epithelium as previously described [3]. Total RNA was purified from the aqueous phase and (1 to 2 µg) used to synthesize double stranded cDNA, for transcription, and quantification of the biotin-labeled cDNA yield using kits from Affymetrix (Santa Clara, CA) [4,5]. An aliquot was used to determine integrity (Agilent Bioanalyzer, Agilent Technologies, Palo Alto, CA) and concentration (NanoDrop ND-1000 spectrophotometer, NanoDrop Technologies, Wilmington, DE).

**Statistical Analyses**

For the univariate analysis, a paired t-test was applied to each probe set to test for differences in mean expression level of the upper and lower small airway epithelium using a Benjamini-Hochberg correction [6]. For the multivariate analyses, three approaches were applied, including: (1) hierarchical clustering approach using average linkage with either Pearson or a Spearman correlation measure (GeneSpring version 7.3, Affymetrix Microarray Suite Version 5 [7]) where the number of times the upper and lower sample of an individual clustered together was counted; (2) a principal component analysis (PCA); and (3) an analysis of the distribution of multivariate, euclidean distance between the upper and lower sample pairs as measured by vector length.

The hierarchical clustering was applied to the probe sets in the “smoking-responsive list” (previously published [8] 529 probe sets, corresponding to 372 unique genes). In each case, the number of times each individual sample pair clustered together was counted, i.e., whether the same individual upper and lower lobe samples clustered most closely with each other. To assess whether there was more cluster pairing in the smoking-responsive list than expected at random, a statistical test of cluster pairing was performed for the smoking-responsive gene list by applying the hierarchical clustering to 100 lists of 529 probe sets selected at random from the list of probe sets present in at least 20% of the upper or lower lobe samples, minus the 529 smoking-responsive list (“100 random lists”). We then clustered each random list and counted sample pairings. Using the distribution of number of pairs for all 100 random lists, we then determined the probability that the smoking-responsive pairing number would have been observed by chance, using a one-tailed approach to determine the p value.

A PCA was applied to the 529 smoking-responsive list. For each, the samples projected on the first three principal components were plotted to assess whether there was strong clustering of upper and lower lobe samples or whether upper or lower pairs clustered together in principal component spaces.

To take into account individual variability, a statistic was generated that compared over-all variability between each upper-lower lobe sample pair. To accomplish this, the length of the vector connecting the upper and lower lobe samples for each individual was calculated for a given set of probe sets, where each probe was mean-centered and scaled by its standard deviation. The vector length was then calculated for each individual, considering the smoking-responsive list, and for the 100 random lists.

Supplemental Data References

1. Coxson HO, Rogers RM, Whittall KP, D'yachkova Y, Pare PD, et al. (1999) A quantification of the lung surface area in emphysema using computed tomography. Am J Respir Crit Care Med 159: 851-856.

2. Perez A, Coxson HO, Hogg JC, Gibson K, Thompson PF, et al. (2005) Use of CT morphometry to detect changes in lung weight and gas volume. Chest 128: 2471-2477.

3. Harvey BG, Heguy A, Leopold PL, Carolan BJ, Ferris B, et al. (2007) Modification of gene expression of the small airway epithelium in response to cigarette smoking. J Mol Med 85: 39-53.

4. Raman T, O'Connor TP, Hackett NR, Wang W, Harvey BG, et al. (2009) Quality control in microarray assessment of gene expression in human airway epithelium. BMC Genomics 10: 493.

5. Tumor Analysis Best Practices Working Group (2004) Expression profiling--best practices for data generation and interpretation in clinical trials. Nat Rev Genet 5: 229-237.

6. Benjamini Y, Hochberg Y (1995) Controlling the false discovery rate: a practical and powerful approach to multiple testing. J R Stat Soc B57: 289-300.

7. Team RDC (2008) R: A language and environment for statistical computing. In: Team RDC, editors. R Foundation for Statistical Computing. Vienna, Austria.

8. Strulovici-Barel Y, Omberg L, O'Mahony M, Gordon C, Hollmann C, et al. (2010) Threshold of biologic responses of the small airway epithelium to low levels of tobacco smoke. Am J Respir Crit Care Med 182: 1524-1532.
